# Alteration of neurofilament heavy chain and its phosphoforms reveals early subcellular damage beyond the optic nerve head in glaucoma

**DOI:** 10.3389/fneur.2023.1091697

**Published:** 2023-03-22

**Authors:** Lan Zhou, Dongyue Lin, Guihua Xu, Xiaoyi Wang, Zilin Chen, Dingding Wang, Huiya Fan

**Affiliations:** ^1^Ophthalmological Center of Huizhou Central People's Hospital, Huizhou, Guangdong, China; ^2^State Key Laboratory of Ophthalmology, Zhongshan Ophthalmic Center, Sun Yat-sen University, Guangzhou, Guangdong, China

**Keywords:** neurofilament heavy chain, optic nerve head, retinal ganglion cells, axon degeneration, acute angle closed glaucoma, chronic angle closed glaucoma

## Abstract

**Background:**

Retinal ganglion cells (RGCs) axon loss at the site of optic nerve head (ONH) is long believed as the common pathology in glaucoma since different types of glaucoma possessing different characteristic of intraocular pressure, and this damage was only detected at the later stage.

**Methods:**

To address these disputes and detect early initiating events underlying RGCs, we firstly detected somatic or axonal change and compared their difference in acute and chronic phase of primary angle-closed glaucoma (PACG) patient using optical coherence tomography (OCT), then an axonal-enriched cytoskeletal protein neurofilament heavy chain and its phosphoforms (NF-H, pNF-H) were utilized to reveal spatio-temporal undetectable damage insulted by acute and chronic ocular hypertension (AOH, COH) in two well characterized glaucoma mice models.

**Results:**

In clinic, we detected nonhomogeneous changes such as ONH and soma of RGCs presenting edema in acute phase but atrophy in chronic one by OCT. In AOH animal models, an increase expression of NF-H especially its phosphorylation modification was observed as early as 4 h before RGCs loss, which presented as somatic accumulation in the peripheral retina and at the sites of ONH. In contrast, in microbeads induced COH model, NF-H and pNF-H reduced significantly, these changes firstly occurred as NF-H or pNF-H disconnection at ONH and optic nerve after 2 weeks when the intraocular pressure reaching the peak; Meanwhile, we detected aqueous humor pNF-H elevation after AOH and slight reduction in the COH.

**Conclusion:**

Together, our data supports that early alteration of NF-H and its phosphoforms would reveal undetectable subcellular damage consisting of peripheral somatic neurofilament compaction, impaired axonal transport and distal axonal disorganization of cytoskeleton beyond the ONH, and identifies two distinct axonal degeneration which were Wallerian combination with retrograde degeneration in acute PACG and retrograde degeneration in the chronic one.

## Introduction

Glaucoma is the leading cause of irreversible blindness worldwide. It consists of different subtypes, including primary angle-closed glaucoma (PACG) and primary open-angle glaucoma (POAG), and among these subtypes, In China, PACG is the most prevalent subtype and is highly deleterious to visual function (1, 2). Elevated intraocular pressure (IOP) is a well-known risk factor of PACG, which leads to an elevated pressure gradient across the lamina cribrosa, predisposing the retinal ganglion cell (RGC) axon to damage at the sites of the optic nerve head (ONH) (3, 4). Clinically, structural measurements of the retinal nerve fiber layer (RNFL) at the ONH are widely used to assess RGC damage (5). However, these structural changes are only detected by current methods when the visual field is seriously damaged (6). The early pathogenesis underlying RGC damage still remains poorly understood. A better understanding of early glaucomatous axon damage is, therefore, essential to develop effective methods, as well as therapeutic agents for the diagnosis and treatment of glaucoma.

To date, evidence from experimental glaucoma models has demonstrated axonal transport deficit at the site of the ONH and the distal visual central system occurs preceding structural loss (7, 8). However, the axonal transport is visualized mostly by virtue of an exogenous anterograde or retrograde tracer; therefore, evidence supporting axonal transport failure at the ONH is circumstantial. The normal function of axonal transport depends on the intact organization of the axonal cytoskeleton, including neurofilaments, microtubules, and their associated proteins, which provides the molecular tracks for the bidirectional movement of motor proteins and their associated cargo (9). Aberrations in several cytoskeletal proteins, such as neurofilament and tau, have been proven to potentially act as surrogate markers for axonal injury and transport damage (10). Among them, neurofilament heavy chain and its phosphoforms have been gaining more and more attention because of their deep involvement in maintaining the integrity of the neuronal cytoskeleton and the important role of revealing early uneasily detectable damage in neurodegeneration diseases (11–14). In glaucoma, the changes in neurofilaments have been investigated in animal models but have remained controversial, as some bodies of research have observed a reduction in neurofilament heavy chain (NF-H) at the ONH in AOH (15, 16); conversely, the elevation of dephosphorylation or phosphorylated NF-H (pNF-H) has been demonstrated in some acute and chronic ocular hypertension (AOH, COH) models (17, 18). This suggests that the changes in NF-H and pNF-H may be different when insulted by different patterns of elevated IOP, and whether alteration of them would indicate early and undetected damage beyond ONH needs further study.

To address these questions, we first evaluated and compared the differences in RGC axonal and somatic changes after an AOH attack and a COH insult in angle-closed glaucoma through an optical coherence tomography (OCT) RNFL and a ganglion cell layer (GCL) scan. Then, from clinic to the basic pathological damage, using two well-characterized glaucoma mice models that mimicked acute and chronic primary angle-closed glaucoma (APACG, CPACG), we attempted to reveal the truths underlying RGC damage through the molecular marker NF-H. In clinic, RNFL and GCL often presented as edema after the acute attack of ocular hypertension in APACG but reduced significantly in CPACG. Our experimental data showed that acute elevated IOP would cause an early increase in phosphorylated NF-H and NF-H and that the accumulation was primarily observed in the soma of peripheral RGCs, while intra-axon swellings and beadings of pNF-H at the ONH site were observed as well. Unlike these features, the expression of pNF-H and NF-H markedly decreased in COH, and these changes first occurred at the ONH and the optic nerve, presenting as local NF-H or pNF-H disconnection. Importantly, we detected a correspondingly significant elevation and a slight reduction in the aqueous humor of both AOH and COH models. Together, our data support the evidence that early alteration of NF-H and its phosphoforms would not only explain the current changes detected by the OCT but also reveal undetectable damage beyond an axonal loss at the ONH, and we identified two distinct characteristics of axonal degeneration that were a retrograde combination with Wallerian degeneration in APACG and chronic dying back degeneration in CPACG.

## Methods

### Classification of patients with glaucoma

Acute primary angle-closed glaucoma (APACG) and CPACG were defined using the International Society of Geographical and Epidemiological Ophthalmology classification (19). The acute attack of APACG is defined by the presence of the following aspects: (1) at least two of the symptoms by the acute episode of IOP increase, which are nausea and/or vomiting, decreased vision, ocular pain or headache, and rainbow-colored halos around lights; (2) IOP ≥ 45 mmHg by the Goldmann applanation tonometry; (3) signs such as corneal epithelial edema, conjunctival injection, shallow anterior chamber, or a mid-dilated unreactive pupil at the first presentation; (4) closed angles in at least three quadrants by a gonioscopic examination; and (5) the start of the symptoms only recently, which were relieved by local or systematic IOP-lowering drugs during hospitalization.

Chronic primary angle-closed glaucoma is demonstrated by the following criteria: (1) visual function damage; (2) at least three quadrants of the posterior trabecular meshwork being invisible on gonioscopy; and (3) gradually elevated IOP without signs of acute angle-closure attack.

The exclusion criteria of the present study were as follows: (1) a previous laser or an intraocular surgery performed on either eye; (2) patients with a subluxated lens or intumescent cataract; (3) patients with uveal effusion; (4) patients with retinal detachment or macular pathology on ocular examination; (5) patients with AL < 19 mm in either eye; (6) both eyes were involved; (7) the pictures or data did not meet the standard for analysis; and (8) basic diseases, such as hypertension or diabetes, are affecting the patient.

### RNFL and ganglion cell layer evaluation through optical coherence tomography

A total of 35 patients with APACG and 30 patients with CPACG aged 50–80 years who visited the glaucoma service of our hospital between January 2020 and December 2021 were recruited for analysis. All patients were administered local or systematic IOP-lowering drugs to eliminate pain and edema, and then, they underwent routine glaucomatous examinations, including the Humphrey visual field, A/B scan and OCT. The RNFL and GCL evaluation was performed by optical coherence tomography angiography (Carl Zeiss AG, Oberkochen, Germany) with two scan models, which were the peripapillary retinal nerve fiber layer and the macular ganglion cell layer. RNFL thickness was measured by reference to a circle scan with a diameter of ~ 3.45 mm, which was divided into four focal sectors: superior, inferior, temporal, and nasal. GCL thickness was acquired with a 6 × 6-mm scanning area centered on the macula. Informed consent was obtained from all patients.

### Animals

We used female or male adult C57BL/6J (6–8 weeks) mice (Vital River, China) for the experiments conducted during the study. All mice were housed under a 12-h light/dark cycle at 22 ± 2°C, with free access to food and water. The animals were habituated in the experimental environment for 1 week before each experimental procedure. All animal experiments were conducted in accordance with the National Institutes of Health Guide for the Care and Use of Laboratory Animals approved by the Institutional Animal Care and Use Committee of Guang Dong Medical University and Zhongshan Ophthalmic Center.

### Acute ocular hypertension and microbead-induced chronic ocular hypertension model

The induction of AOH was performed on the right eye as previously reported (20). Briefly, animals were anesthetized by an intraperitoneal injection of a 1:1 mixture (1.0 ml/kg) of ketamine (100 mg/ml, Fujian Gutian Pharmaceutical Co., Ltd., China) and xylazine (20 mg/ml, Sigma, Germany), the cornea was topically anesthetized with 0.5% tetracaine hydrochloride, and the pupil was dilated with 1% tropicamide. Next, the anterior chamber of the right eye was perfused with a 30-gauge needle connected to a normal saline reservoir, which was elevated to a height of 80 cm H_2_O (60 mmHg) for 2 h. The same operation was performed on the eyes of the sham control group but without increasing the pressure. IOP was confirmed by a tonometer (Tono-Lab; ICARE, Finland). Three different time points, including 4, 6, and 12 h after AOH, were set for histological and molecular studies, while 30 mice were used for analysis (*n* = 5 mice per experiment at each time point) and 4 mice were excluded because of cataract or intra-ocular hemorrhage.

A microbead-induced chronic ocular hypertension model was established as described in earlier sections (21, 22). Before a microbead injection, baseline IOP was acquired using the tonometer (Tono-Lab; ICARE, Finland) in mice anesthetized with 2.5% isoflurane (Minrad Inc., Bethlehem, PA, USA) for at least two consecutive days prior to surgery. Next, 2 μl of 3-μm magnetic microbeads (Bangs Laboratories, Inc, IN, USA) was injected into the anterior chamber angle of the experimental mice anesthetized with 2.5% isoflurane, and the same volume of sterile saline was injected into the anterior chamber of control mice. After the surgery, antibiotic drops (0.5% ofloxacin hydrochloride ophthalmic solution; Alcon Laboratories, Inc., USA) were applied in the surgery eye, and the animal recovered for 24 h prior to IOP measurements. IOP was measured every 2 days for 10 days and then every 5 days for 20 days. Thirty-six mice were prepared for the experiment; six mice with inadequate IOP elevation and infection were excluded. Finally, 20 mice were used for the IOP measurement: nine mice at 2 weeks and the remaining 11 mice housed for 4 weeks were used for a further study.

### Tissue collection

Tissues such as retinal wholemount, sections, and the optic nerve were collected at a certain time point of AOH and COH animal models. A 20-mm cryostat section was acquired from the eye cups embedded in Optimal Cutting Temperature compound (OCT, Tissue-Tek, USA) after being fixed in 4% paraformaldehyde (PFA) for 30 min and dehydrated in 20% sucrose dissolved in PBS overnight. To obtain the retinal wholemount and the optic nerve, mice were transcardially perfused with cold saline followed by 4% paraformaldehyde for 30 min. Their eyes were enucleated, the cornea and the lenses were removed, and the eye cups were postfixed in 4% PFA for 10 min. The retinas or the retinal nerve were then dissected from the eye cups for further study.

### Western blot analysis

The retinas and the optic nerve head from AOH and COH models were harvested at a certain time point and then homogenized in RIPA lysis bu?er (Millipore, Germany). Then, the sample was separated on SDS–PAGE and electroblotted onto a PVDF membrane (Bio-Rad, USA). Primary antibodies against phosphorylated neurofilament-H (1:500; Millipore, Germany), neurofilament-H or L (1:1,000; Millipore, Germany), neurofilament-M (1:1,000; Cell Signaling Technology, USA) followed by incubation with a HRP-conjugated secondary antibody were used. Antibody-reactive bands were visualized using an Immobilon Western Chemiluminescent HRP Substrate (Millipore, Germany) and a G-BOX (Syngene, USA) image capture system. Relative protein expression level was measured by calculating the band density with ImageJ software (NIH, USA), followed by normalization to the GAPDH loading control.

### Collection of the aqueous humor from mice

The aqueous humor was collected from the experiment and control eyes of AOH or COH using a syringe with a 35-gauge needle (Hamilton). A total of 3 μl of AH was collected from each mouse, and 15 samples were pooled for ELISA. The aqueous humor was collected 6 h after AOH and 2 weeks after COH.

### Immunofluorescence staining of retinal wholemount and retinal and optic nerve cryostat sections

To analyze the spatial distribution of NF-H and its phosphoforms, we performed immunofluorescence staining in retinal wholemount and the retinal and optic nerve cryostat sections. After drying and rehydration in PBS, the samples were permeabilized in 0.5% Triton X-100 for 30 min, blocked in 5% donkey serum for 1 h at room temperature (RT), and incubated overnight at 4°C with primary antibody, which was followed by incubation with an Alexa Fluor-labeled secondary antibody (Cell Signaling Technology, Beverly, MA, USA) for 1 h at RT after three washes with PBS. Primary antibodies against phosphorylated neurofilament-H (1:500, Millipore, Germany), Neurofilament-H (1:1,000, Millipore, Germany), Brn3a (1:200, Millipore, Germany), and NeuN (1:500, Millipore, Germany) were used for IF staining. The samples were finally examined under a confocal microscope (LSM710; Carl Zeiss, Germany). Retinal wholemount was dissected into four quadrants, three different eccentricities away from the optic disk in each quadrant: central (0.5 mm), middle (2 mm), and peripheral (4 mm) were chosen for quantification analysis with a 20× objective (500 × 500 μm for each image, 12 images per retina). Five histological sections (400 × 400 μm) through the optic nerve head (centered at ~0.4 mm from the scleral margin) or the optic nerve (centered ~1.2 mm away from the scleral margin) of each IOP elevation and the control eye were examined with a 10 × objective by two masked observers.

### Analysis of neurofilament score

The analysis of neurofilament disconnection was performed using an established scoring system ranging from 0 to 2, which was graded as follows: 0 = intact structure and no retraction bulbs, 1 = shortened axons and some retraction bulbs, and 2 = loss of structural integrity and plenty of retraction bulbs and holes (23). The score was averaged for the final statistical analysis.

### ELISA measurement of the aqueous humor

The aqueous humor collected from AOH and COH models was used for quantifying pNF-H through an enzyme-linked immunosorbent assay (ELISA) kit (Phosphorylated Neurofilament Sandwich ELISA Kit, Merck KGaA, Germany), and the analysis was carried out according to the manufacturer's protocols. The samples were diluted at a ratio of 1:6 with a sample diluent. pNF-H antibody-coated strip wells were incubated with 50 μl of standards or samples at room temperature (RT) for 1 h with gentle shaking. Plates were washed six times with diluted wash buffer provided in the kit; then, 100 μl of detection antibody was added to all wells and incubated for 1 h at RT. Plates were washed six times and 100 μl of the diluted alkaline phosphatase-conjugated goat anti-rabbit polyclonal antibody was added to each well and incubated for 1 h. After six washes, 100 μl of freshly diluted 1× pNPP alkaline phosphatase substrate was added to each well and incubated in the dark at RT for 30–60 min. Stop solution (3N NaOH) was added and plates were read at 405 nm on an Infinite 200 PRO NanoQuant Plate reader (Tecan, Switzerland) using i-control analytical software.

### Statistical analysis

IBM SPSS Statistics 22 (IBM Corp: Armonk, NY, USA) was used for all statistical analyses. For the comparison of clinical data between the glaucoma eye and the contralateral unaffected eye, a two-tailed *t*-test, the Mann–Whitney rank sum test, and Pearson's chi-square (χ^2^) test were used to obtain for a variety of data. Differences in IOP throughout the 4-week experimental time course between the COH and the control groups were statistically analyzed using a one-way repeated-measures analysis of variance (ANOVA). For neurofilament expression, a comparison between all experimental groups at each time point was carried out using one-way analysis of variance (ANOVA) followed by a Dunnett's test for normally distributed data and equal variances; otherwise, it was analyzed using the Kruskal–Wallis analysis followed by the Dunn–Bonferroni *post hoc* method. Differences in pNF-H accumulation, neurofilament disconnection between glaucoma and the control eye were assessed with a two-tailed *t*-test. Graphical representations of the data were made with scatter diagrams or box-and-whisker plots. A *P*-value of < 0.05 was considered significant.

## Results

### In clinic, RGC axon and soma exhibited signs of edema after acute attack in APACG but presented a significant degeneration insulted by chronic IOP elevation in CPACG

**Figure 1:**
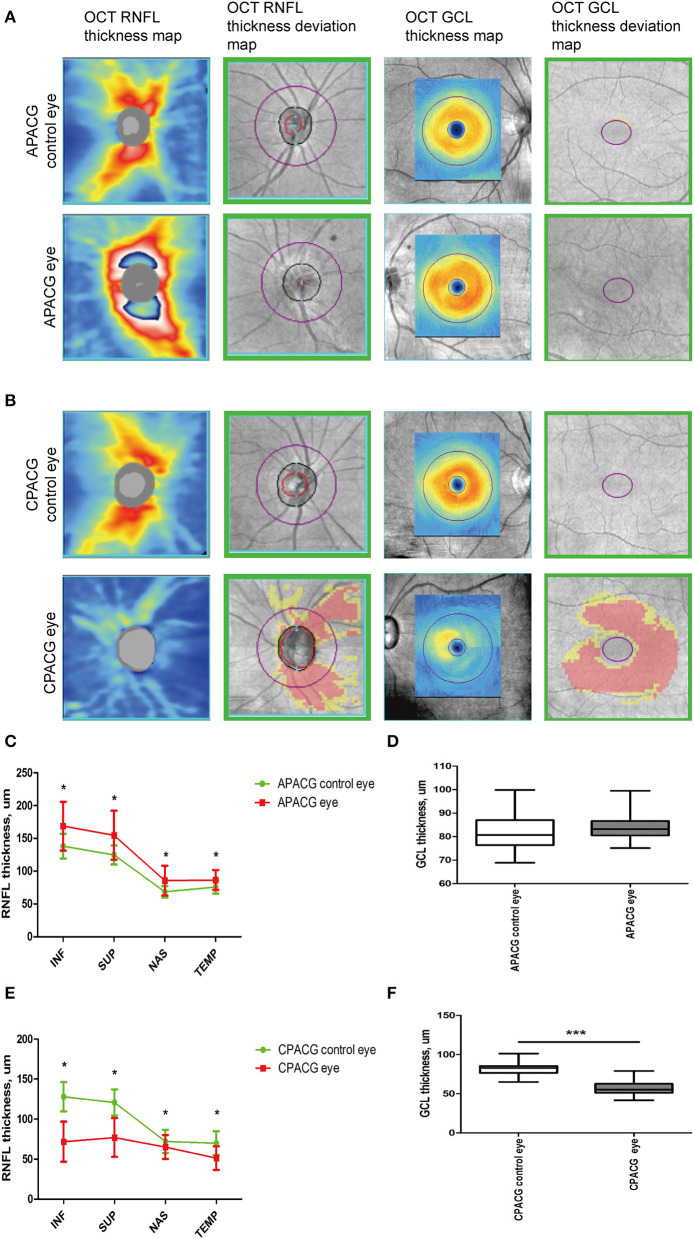
Heterogeneity such as RGC axon and soma presenting edema insulted by acute damage but atrophy in chronic glaucoma evaluated by OCT. **(A)** OCT RNFL thickness maps, OCT RNFL thickness deviation maps, OCT GCL thickness maps, and OCT GCL thickness deviation maps are presented, which show that the thickness of RNFL and GCL increased significantly after an acute attack. The warm colors on the thickness maps represent a thicker RNFL or GCL and cooler colors represent a thinner one. **(B)** OCT imaging showed an obvious loss of RNFL and GCL loss insulted by chronic damage in CPACG. The cooler colors in thickness maps suggested RNFL and GCL thickness reduction. Areas coded in red and yellow in deviation maps represent measurements below the 99th and 95th percentile ranges, respectively. **(C)** Peripapillary RNFL thickness was quantified in four regions, including the inferior (INF), superior (SUP), nasal (NAS), and temporal (TEP) regions. The line graph indicates that the detailed RNFL thickness of patients with APACG (colored in red) reduced significantly, with the contralateral unaffected eyes as the control group. *P < 0.05. **(D)** The bar graph shows the average GCL thickness in patients with APACG. GCL thickness increased slightly. Data are presented as mean ± SD. **(E)** The line graph shows that the RNFL thickness in patients with CPACG reduced significantly, and a Mann–Whitney rank sum test was used to analyze the data, *P < 0.05. **(F)** Macular GCL thickness in CPACG was obviously reduced. Data are presented as box-and-whisker plots, where the “box” delineates the median and the 25th and 75th quartiles and the “whiskers” show the minimum and maximum values. A two-tailed *t*-test was used to analyze the data, ***P < 0.001.

Retinal nerve fiber layer and GCL thickness in OCT are taken as important tools to evaluate RGC damage in clinic. To determine the changes in RGCs insulted by acute or chronic ocular hypertension in clinic, RNFL and GCL scans of OCTA were performed in APACG and CPACG. Thirty-five patients with APACG and 30 patients with CPACG were included in the analysis. The contralateral eyes of each group without glaucoma were set as control. Demographic data for APACG and CPACG are shown in Table 1. There were no significant differences in age or sex between the two groups (Table 1). Peripapillary RNFL thickness localized at the four sectors in APACG eyes increased significantly compared to control eyes (inferior: 168.54 ± 37.08 vs. 138.11 ± 18.94 μm, superior: 154.74 ± 37.45 vs. 124.74 ± 14.59 μm, nasal: 85.57 ± 22.71 vs. 68.37 ± 8.64 μm, and temporal: 86.43 ± 15.17 vs. 75.8 ± 10.28 μm; *P* < 0.05, Figures 1A, C) after an acute attack. The average thickness of GCL also showed to be slightly elevated in APACG vs. control (84.16 ± 5.19 vs. 81.32 ± 7.30 μm, *P* = 0.067, Figures 1A, D). By contrast, RNFL thickness maps and thickness deviation maps in CPACG vs. control eyes showed an obvious loss of RNFL within four sectors of the optic disk (inferior: 71.80 ± 25.07 vs. 127.97 ± 18.23 μm, superior: 76.97 ± 24.30 vs. 120.77 ± 16.39 μm, nasal: 65.17 ± 14.95 vs. 72.13 ± 14.45 μm, and temporal: 51.23 ± 14.64 vs. 72.13 ± 14.45 μm, *P* < 0.05, Figures 1B, E). The GCL thickness also reduced significantly in CPACG vs. control (57.55 ± 9.32 vs. 81.91 ± 7.37 μm, *P* < 0.001, Figures 1B, F). Taken together, the data from clinical patients indicated that RGC axon and soma presented edema after the acute attack in APACG but degeneration in CPACG, which suggested that two types of RGC damage existed between them.

**Table 1:** Demographics and basic clinical data of recruited eyes.

		**APACG**			**CPACG**			***P*** **-value**
		**Affected eye**	**Contralateral eye**	***P*** **-value**	**Affected eye**	**Contralateral eye**	***P*** **-value**	
Age	Mean ± SD	62.3 ± 7.1		-	60.1 ± 7.8		-	0.23^a^
	Range	46–84			43–76			
Sex	Male	7		-	11		-	0.13^c^
	Female	28			19			
IOP (mmHg)	Mean ± SD	51.0 ± 7.6	15.4 ± 3.5	<0.001^a^	34.7 ± 6.5	15.5 ± 3.8	<0.001^a^	-
	Range	34–62	10–25		26–54	9–24		
VA, LogMAR	Median	0.5	0.2	<0.001^b^	0.5	0.1	<0.001^b^	-
	Range	0.2–3.3	0–0.5		0.2–3.6	0–0.5		

Values are mean ± SD for continuous factors, median for visual acuity (VA).

a Student's t-test.

b Mann–Whitney U-test.

c Pearson χ^2^ test.

### Anterior chamber perfusion with normal saline in mice to mimic acute attack of APACG and a microbead injection in the anterior chamber angle to mimic chronic IOP elevation of CPACG

**Figure 2:**
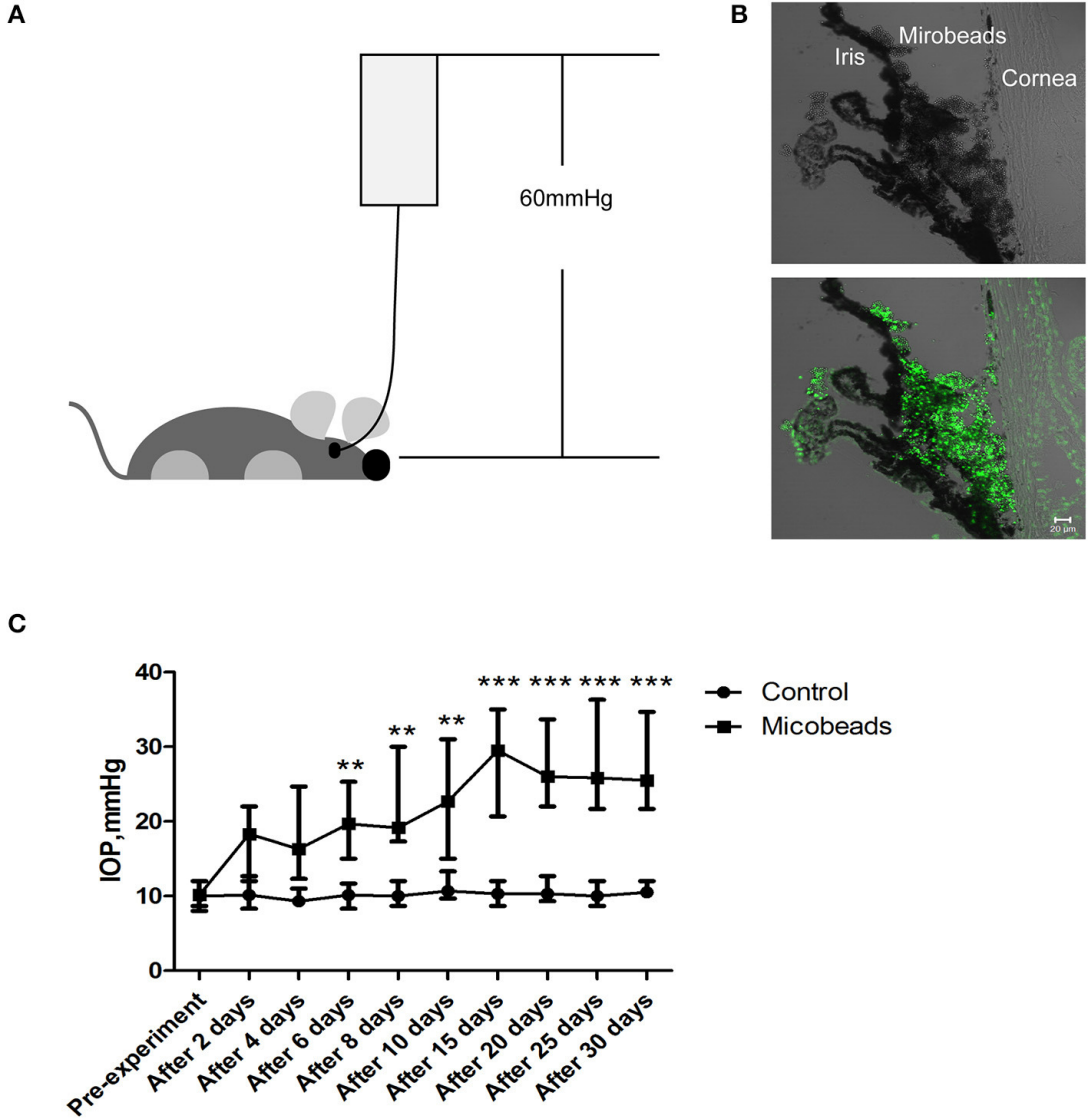
Two well-established glaucoma animal models were set to mimic acute attack of APACG and chronic damage of CPACG. **(A)** Acute ocular hypertension model: IOP acutely increased to 60 mmHg by perfusion with normal saline into the anterior chamber, and the ocular hypertension was sustained for 2 h. **(B)** Three-micrometer magnetic microbeads were injected into the anterior chamber angle to mimic chronic ocular hypertension. The microbeads showed autofluorescence (bottom). Scale bars: 20 μm. **(C)** IOP began to increase after 2 days and reached the peak 2 weeks after the injection, IOP was measured at pre-treatment, 2, 4, 6, 8, and 10 days, and then every 5 days for 1 month after the surgery. *n* = 20 mice, **P < 0.01, ***P < 0.001.

Next, to uncover what lies beneath the aforementioned clinical observations, two animal models were set to mimic the acute attack of APACG and chronic IOP elevation of CPACG. In AOH animal models, the IOP immediately increased to 60 mmHg by perfusion with normal saline into the anterior chamber, which well–simulated the state of onset in APACG (Figure 2A). In the microbead-induced models, the IOP began to increase at 2 days (average IOP, 17.43 ± 3.06 mmHg vs. control 10.13 ± 1.12 mmHg; *P* < 0.01) and reached the peak at 2 weeks (28.10 ± 5.42 mmHg vs. control 10.30 ± 1.02 mmHg, *P* < 0.001) after the microbead injection into the anterior chamber angle (Figures 2B, C). The elevation of IOP sustained for 1 month and began to decrease (Figure 2C).

### Acute ocular hypertension caused early elevation of phosphorylated NF-H in the retina and the ONH, and an evident neurofilament increase in the retina

Neurofilaments are major proteins synthesized by RGCs and conveyed along the ON by fast axonal transport. Alterations in neurofilaments, especially NF-H and its phosphoforms, had been proven as biomarkers of axonal transport failure in some neurodegenerative diseases (24, 25). However, other studies from traumatic brain injury showed that intra-axonal neurofilaments' compaction may occur independent of axonal transport failure and not evoke axonal swelling (11). Since our clinical observation from APACG only showed neuronal swelling, which encouraged us to question whether it was caused by neurofilaments' compaction or transport failure, we performed the spatiotemporal analysis in the aforementioned animal models.

**Figure 3:**
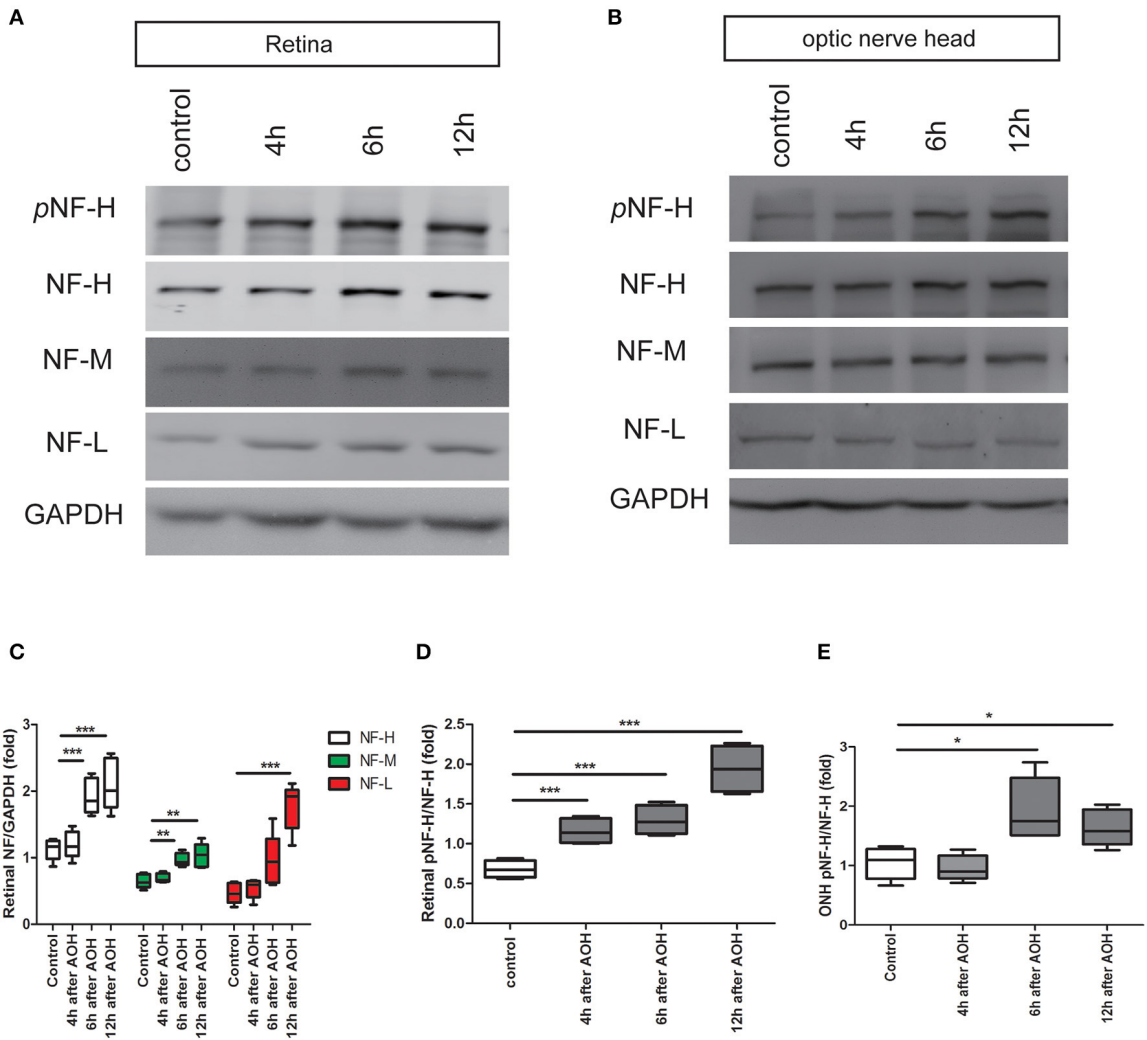
Early elevation of pNF-H expression observed in the retina and ONH following induction of AOH. An obvious elevation of the neurofilament was also observed in the retina. **(A)** Representative blots of NF-H, M, L, and pNF-H from AOH and the control retinas at 4, 6, and 12 h are shown. In the retina, the bands of the neurofilament three subunits were more visible at 6 and 12 h, while the band of pNF-H was more visible as early as 4 h. pNF-H, NF-H was recognized at 200 kDa, NF-M was visible at 160 kDa, and NF-L was at 70 kDa. GAPDH was used as an internal control. **(B)** The expression of neurofilament subunits and pNF-H in the ONH was traced at 4, 6, and 12 h after AOH, but western blotting of the ONH showed that NF-H, NF-M, and NF-L were unchanged, while the pNF-H band increased significantly at 6 h. **(C)** Densitometry measurements of three neurofilament subunits in the retina (normalized for GAPDH and expressed relative to the control) are provided in the box-and-whisker plots, *n* = 5 mice for each group, **P < 0.01, ***P < 0.001. **(D, E)** Quantification of pNF-H expression from the retina at 4 h and ONH at 6 h is shown as box-and-whisker plots, respectively. *n* = 5 mice for each group, *P < 0.05, ***P < 0.001.

Our previous study has demonstrated that apoptotic RGCs and axon loss were firstvisualized as early as 6 h after AOH (20). Based upon these, we detected the expression of neurofilament and its phosphoforms in the retina and ONH, respectively, from 4 h after AOH preceding RGC apoptosis and axon loss. Neurofilament subunit including NF-H, M, and L increased significantly in the retina after 6 h; among them, NF-H was elevated by almost two-fold compared to control, which was the most obvious one (1.92 ± 0.26 vs. control 1.12 ± 0.16, *P* < 0.001, Figures 3A, C). More importantly, retinal pNF-H levels showed a significant increase as early as 4 h after AOH (pNF-H/NF-H, 1.16 ± 0.16 vs. control 0.68 ± 0.11, *P* < 0.001) when the neurofilament did not change (Figures 3A, D). For the tissues of ONH, we did not detect significant changes in neurofilament subunits at an early time (4 h) when the retinal pNF-H began to increase; however, we detected a nearly two-fold increase of pNF-H at 6 h after AOH (pNF-H/NF-H, 1.94 ± 0.53 vs. control 1.04 ± 0.27, *P* < 0.05, Figures 3B, E). Thus, our data showed an early elevation of NF-H and its pNF-H in the retina and only a significant increase of pNF-H in the ONH.

### NF-H and its phosphoforms accumulated in the soma of peripheral RGCs after AOH

**Figure 4:**
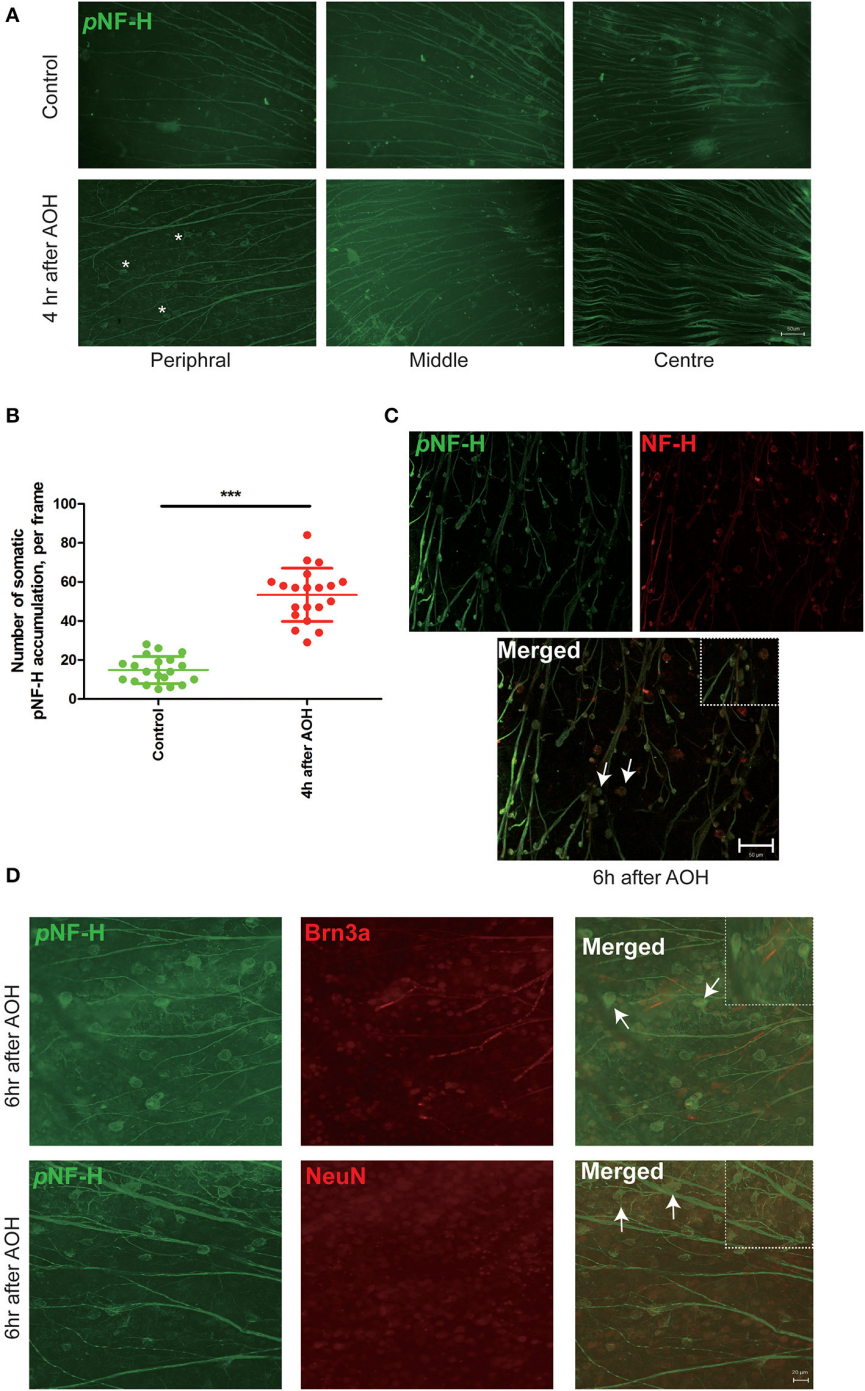
NF-H and its phosphoforms accumulated in the soma of peripheral RGCs after AOH. **(A)** Immunofluorescence of the pNF-H marker, SMI31, in the retinal wholemount 4 h after AOH; retinal wholemount was observed from peripheral to central regions. Asterisk indicates pNF-H accumulation in the peripheral retina. Scale bars: 50 μm. **(B)** Quantification of pNF-H accumulation in the peripheral retina. The data are presented as a scatter diagram. Mean ± SD is shown, *n* = 5 for each group, ***P < 0.001. **(C)** Immunofluorescence of pNF-H and NF-H in the retinal wholemount of AOH after 6 h. Micrographs show that most of the pNF-H accumulations were overlapped with NF-H, with arrows indicating examples of co-expression, and the high magnification image is also shown (top right of the third picture), Scale bars: 50 μm. **(D)** Immunofluorescence of pNF-H and the RGC markers Brn3a and NeuN in the retinal wholemount of AOH after 6 h. pNF-H accumulations were highly overlapped with an RGC marker, and the high magnification images are also shown (top right of the third picture and sixth picture). Scale bars: 20 μm.

Based on the changes in NF-H and its phosphoforms' expression in the Western blot, the retinal wholemounts were collected to study their distribution through immunofluorescence staining. In the retina, an abnormal increase of NF-H and its pNF-H presented as a character of protein deposits or accumulations in the cell bodies along the axon, which first appeared at 4 h after AOH in the peripheral retina (asterisk, Figure 4A). After 6 h of AOH, the NF-H also began to accumulate, and the accumulation of pNF-H and NF-H was highly overlapped (Figure 4C). The number of somatic pNF-H deposits was then counted, and we found that it increased remarkably in the AOH group (53 ± 14 vs. control 14 ± 7 per field, *P* < 0.001, Figure 4B). Then, to confirm that pNF-H accumulation was located in the soma of RGCs, several cell markers, including Brn3a and NeuN, were used for immunofluorescence staining. pNF-H deposits were highly co-labeled with these two markers (Figure 4D), indicating that pNF-H and NF-H accumulated in the soma of peripheral RGCs.

### At the sites of ONH, pNF-H exhibited a sign of intra-axon plaque and vacuolization after acute attack

**Figure 5:**
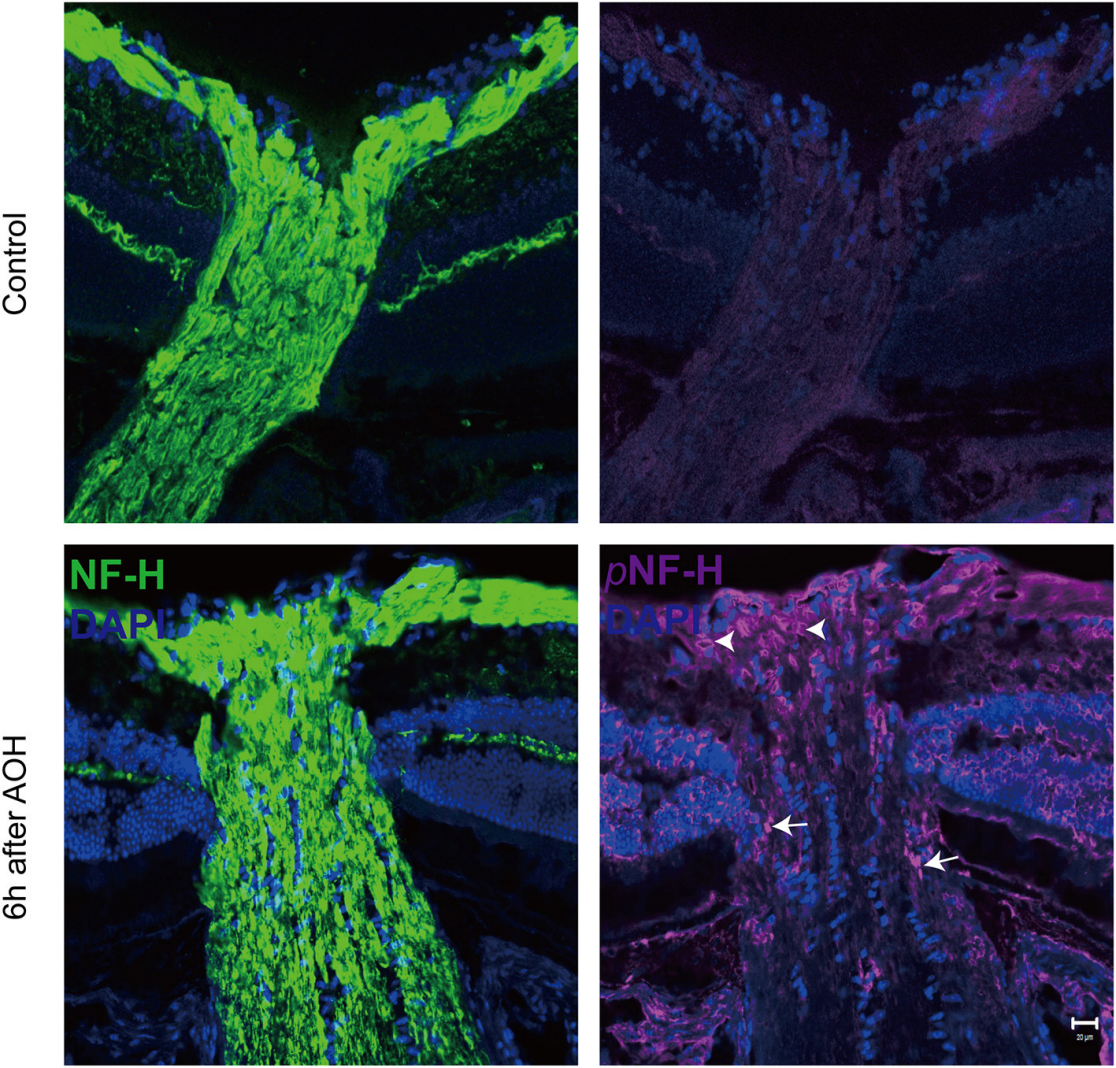
Phosphorylated neurofilament heavy chain (pNF-H) presented as intra-axon plaque and vacuolization at the site of ONH after an acute attack. Immunofluorescence of pNF-H and NF-H in the ONH 6 h after AOH. The arrowhead shows the vacuolization, while the arrows show the plaque. Scale bars: 20 μm.

Optic nerve cryostat sections were harvested to study the spatial distribution of pNF-H since we found that its expression increased at the ONH. The ONH has been considered as the primary site of early axonal cytoskeleton damage. However, the neurofilament including NF-H, M, and L here did not show significant change at an early time (Figure 3B). At 6 h after AOH, NF-H phosphoforms began to accumulate at the ONH, which exhibited a sign of intra-axon plaque (Figure 5, arrows) and vacuolization (Figure 5, arrowheads) different from their accumulation in RGC soma.

### Chronic ocular hypertension led to a marked reduction in pNF-H and NF-H at an early time

**Figure 6:**
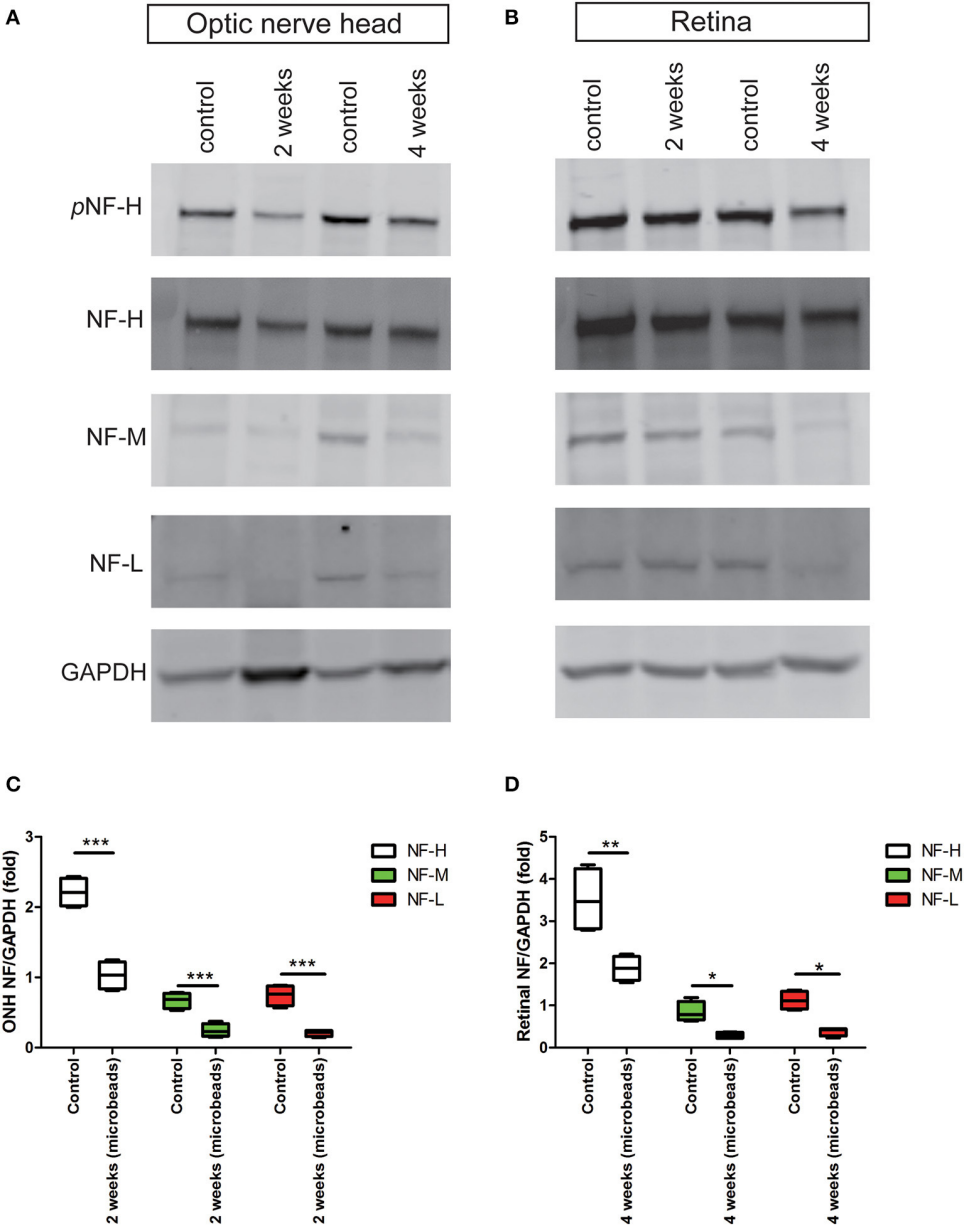
Early reduction in pNF-H and NF-H expression in the ONH after chronic ocular hypertension. **(A)** Western blot showing control and treated ONH 2 and 4 weeks after COH occurred. pNF-H and NF-H was recognized at 200 kDa, NF-M was visible at 160 kDa, and NF-L was visible at 70 kDa, with GAPDH as the internal control. The density of pNF-H and neurofilament subunit bands started to reduce at 2 weeks. **(B)** Representative blots from control and treated retinas 2 and 4 weeks after COH occurred. The density of pNF-H and NF-H began to reduce 4 weeks after COH occurred. **(C, D)** Densitometry measurements of three neurofilament subunits in the ONH and the retina (normalized for GAPDH and expressed relative to the control) are provided in the box-and-whisker plots, *N* = 4 for each group, *P < 0.05, **P < 0.01, ***P < 0.001.

The accumulation of pNF-H at the site of peripheral RGCs and ONH induced by AOH prompted us to observe the changes induced by the COH insult. Therefore, the expression of pNF-H and NF-H had been analyzed in different tissues of the COH model. However, unlike the alteration in AOH, a chronic IOP elevation caused a significant reduction in neurofilament and NF-H phosphoforms at the ONH as early as 2 weeks when the IOP reached the peak after a microbead injection (NF-H/GAPDH: 1.03 ± 0.20 vs. control 2.21 ± 0.21, *P* < 0.001, Figures 6A, C). In the tissues of the retina, the expression of neurofilament, especially NF-H or pNF-H, did not change till 4 weeks, after which it began to reduce (NF-H/GAPDH: 1.88 ± 0.29 vs. control 3.51 ± 0.77, *P* < 0.001, Figures 6B, D). In general, the data here suggested that axonal cytoskeleton damage occurred first in the ONH in COH.

### Neurofilament degeneration first occurred at ONH and the optic nerve presented as local NF-H or pNF-H disconnection

**Figure 7:**
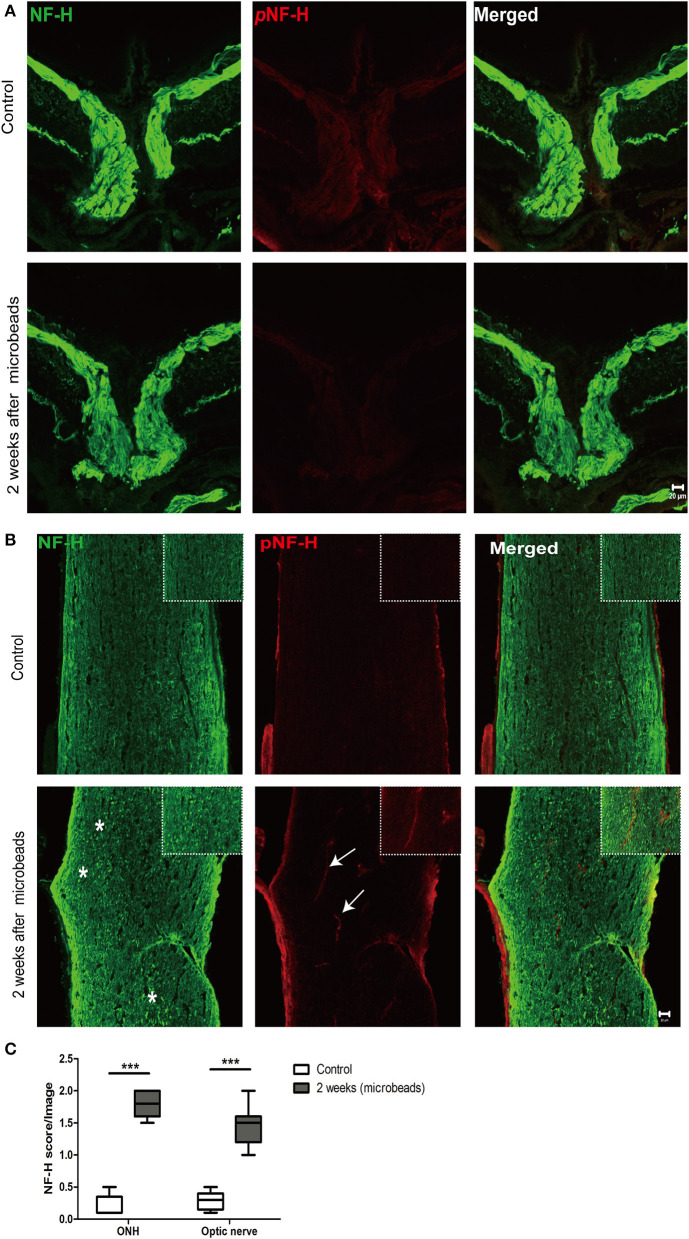
Neurofilament degeneration first occurred at ONH and the optic nerve presented as local NF-H or pNF-H disconnection. **(A)** Feature double labeling immunofluorescence of pNF-H with NF-H at the site of ONH showing general neurofilament reduction and disconnection at the ONH after 2 weeks, Scale bars: 20 μm. **(B)** Longitudinal optic nerve cryostat sections were harvested and used for immunohistochemistry of pNF-H and NF-H. Two weeks after COH, the neurofilament showed disconnection and dispersed into granular ones (asterisk), with arrows showing local pNF-H disconnection; high magnification images are shown in the top right of each lower magnification picture. Scale bars: 20 μm. **(C)** An established scoring system was used for the quantification of neurofilament disconnection. The detailed score is shown in the box-and-whisker plots and a higher score is displayed in COH. *N* = 5 for each group, ***P < 0.001.

To further confirm the characteristic of neurofilament damage, we performed immunofluorescence staining of NF-H and pNF-H in the longitudinal ONH and optic nerve cryostat sections. The pattern of NF-H at the sites of ONH showed a significant filament disconnection where the neurofilaments were disrupted and detected retraction bulbs or loss of structural integrity by COH (Figure 7A). The extent of ONH NF-H disconnection was calculated using an established scoring system. The results revealed that COH caused almost four-fold greater damage compared to the control by 2 weeks (1.7 ± 0.4 vs. 0.2 ± 0.1, *P* < 0.001, Figures 7A, C). Of particular concern was the NF-H disconnection at the optic nerve, whereby it dispersed into granular ones after COH (asterisk, Figure 7B) and the NF-H score increased significantly compared to the control (1.4 ± 0.3 vs. 0.3 ± 0.1, *P* < 0.001, Figure 7C). For pNF-H immunofluorescence staining, besides a presentation of general reduction at the ONH and the optic nerve, it also showed a local vessel-like structural debris disconnection in the degenerated axon. Therefore, the data here suggested that COH caused early neurofilament damage at the ONH and that the optic nerve presented as local NF-H or pNF-H disconnection.

### pNF-H was elevated in the aqueous humor of AOH but reduced in the aqueous humor of COH

**Figure 8:**
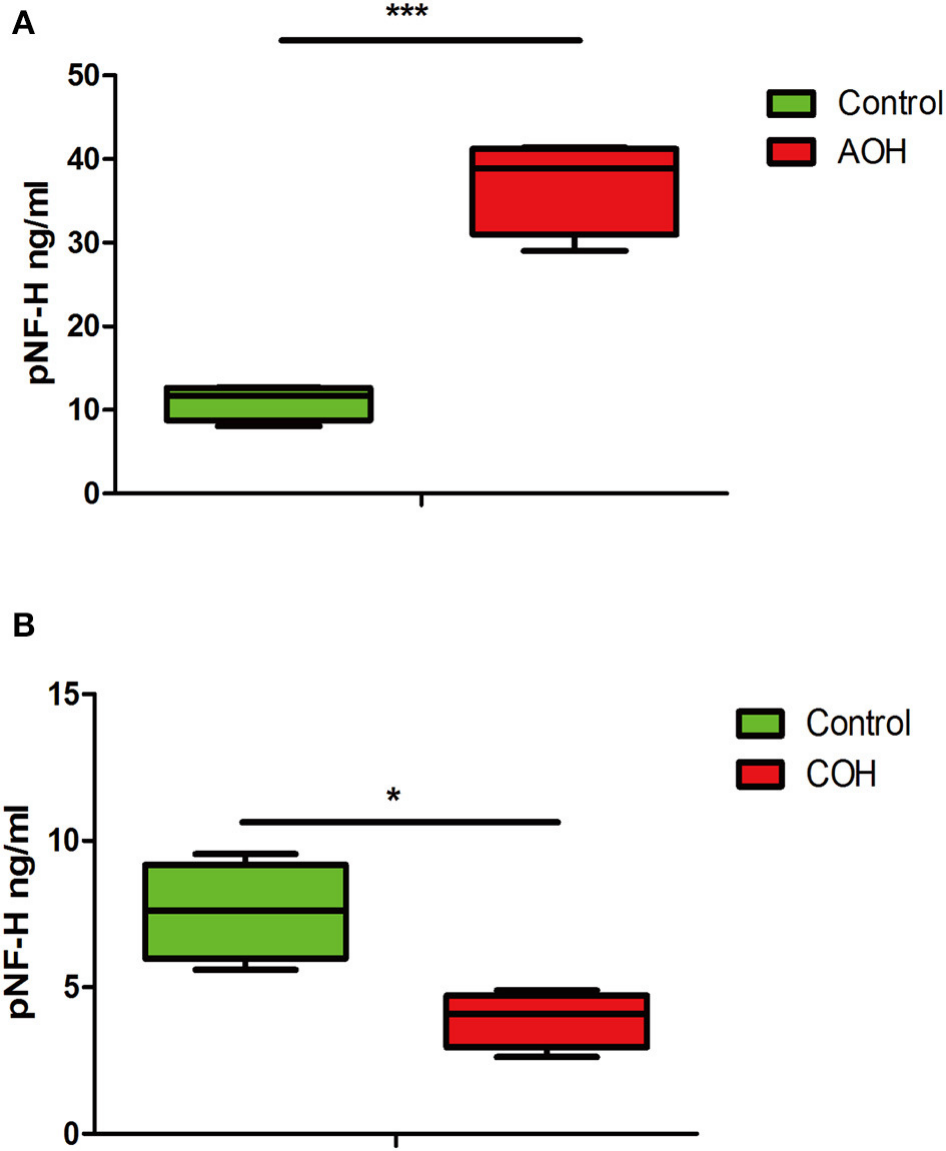
Corresponding aqueous humor pNF-H elevation in AOH and reduction in COH were observed. **(A)** Quantification of pNF-H concentration by ELISA in the aqueous humor at 6 h after AOH. **(B)** Quantification of pNF-H concentration by ELISA in the aqueous humor at 4 weeks of COH. All the data are presented as box-and-whisker plots, where the “box” delineates the median and the 25th and 75th quartiles and the “whiskers” show the minimum and maximum values, *n* = 15 for each group, ^*^P < 0.05, ^***^P < 0.00.

The phosphorylated forms of NF-H have been proven to be released from damaged and diseased axons and are particularly resistant to proteolytic cleavage (12, 13, 26). Therefore, to evaluate the pNF-H change released from damaged RGCs of the aforementioned experimental glaucoma models, we measured the pNF-H concentration by ELISA in the aqueous humor collected from AOH and COH animal models. The results showed that pNF-H was significantly elevated in the aqueous humor of AOH vs. control (37.03 ± 5.71 vs. 11.06 ± 2.15 ng/ml*, P* < 0.001, Figure 8A). By contrast, in COH, the concentration of NF-H in the aqueous humor reduced slightly (3.93 ± 0.95 vs. 8.17 ± 1.66 ng/ml, *P* < 0.05, Figure 8B). Our results here showed that pNF-H change in the aqueous humor took place corresponding to its alterations in the damaged RGCs insulted by AOH and COH.

## Discussion

In the current study, we introduced the molecular marker NF-H and its phosphoforms to study early subcellular damage in glaucoma and explain the heterogeneity detected in patients with clinical glaucoma. Our results suggested that the ONH was not the only primary damage site when insulted by different durations and degrees of elevated IOP in angle-closed glaucoma. When attacked by AOH, beside NF-H and pNF-H intra-axonal plaque or vacuolization at the ONH, their accumulation in the peripheral RGC soma was demonstrated to be early damage, which was also the potential pathological damage beneath RNFL or GCL edema in a patient with APACG. Furthermore, after being insulted by COH, focal intra-axonal NF-H and its phosphoforms' loss or disconnection at the site of ONH and the optic nerve proved to be the primary damage event, which also explained why the axonal degeneration often occurred in the OHN. Meanwhile, the two different types of damage suggested here that the neurons in AOH were degenerated by Wallerian and retrograde degeneration, while those in COH were degenerated by retrograde degeneration.

The lamina cribrosa of the ONH has long been believed to be the primary site of RGC damage in glaucoma. Pioneering basic research performed on monkeys (27–29) and pigs (30) insulted by AOH and COH has demonstrated axonal transport failure preceding axonal loss at the lamina cribrosa. Rodents lack a true lamina cribrosa, but their ONH owns a similar function as the lamina cribrosa. Similar operations performed on rodents have revealed that the ONH is the site of early axonal transport disruption and cytoskeleton damage (7, 31, 32). In clinic, it is difficult to monitor the changes in axonal transport directly. With the use of OCT, the peripapillary RNFL analysis is considered as one important tool for detecting the changes in the entire RGC axon at the ONH, and RNFL reduction is considered as one of the necessary criteria for the diagnosis of glaucoma (33). However, the RNFL analysis only represents the structural changes in the ONH. Furthermore, the point of emphasis too much on the pathological changes of OHN would exclude the subcellular changes in RGCs at other sites. Homogeneity or variability of RNFL changes in the ONH was also found in our clinical data, whereby even when insulted by an acute elevated IOP (above 50 mmHg) for hours or days, we did not detect classical RNFL atrophy in these patients. Conversely, RNFL thickness and somatic atrophy were often observed simultaneously in patients suffering from chronic elevated IOP. It thus raised two questions: Is ONH the common primary damage site in glaucoma? What happens when intra-RGCs are insulted by different degrees and durations of elevated IOP? In fact, recent studies increasingly focused on cellular changes beyond ONH (6). A proteomic analysis of the experimental glaucoma retinas demonstrated that the cytoskeletal protein is the most obvious and earliest changed molecule (34, 35). Urged by these facts, we performed a histological study using a major cytoskeletal protein neurofilament in different tissues of glaucoma animal models. Our study revealed that the primary damage site in angle-closed glaucoma varies depending on the character of the initial stressor (elevated IOP); in AOH, accumulation of pNF-H and NF-H in the peripheral RGC soma suggests that the peripheral retina would be affected as well; in COH, the early disconnection of NF-H in the ONH and the optic nerve indicated that damage first appears in the distal optic nerve and progresses to the proximal retina. Therefore, our study helps to strengthen an important viewpoint that the primary damage site in glaucoma is not limited to the ONH.

Neurofilament is one of the dominant proteins of the axonal cytoskeleton in RGCs, which is composed of triplet subunits, a light (NF-L), a medium (NF-M), and a heavy (NF-H) chain. Neurofilaments are first generated in the soma of RGC and concentrated in the axon, where they play an important role in maintaining the extreme morphology of RGCs (12). Among them, the protein of NF-H has aroused great interest because of its very unusual properties. First, it contains 50–60 back-to-back hexa, hepta, or octapeptide repeats, each containing the sequence Lysine-Serine-Proline (KSP); the serine residues in these peptide repeats can be highly phosphorylated after the protein is transported from the neuronal cell soma to the axon (12, 36). Second, the phosphorylated forms of NF-H are quite resistant to proteases. Once this protein is hyperphosphorylated, it would lead it to accumulate and form aggregates that ultimately block axonal transport (24, 37). The properties of this protein suggest that it would be used as a sensitive marker to indicate early change of damaged or diseased axons. As recently shown, accumulation of pNF-H has been observed in a range of neurodegenerative diseases, including amyotrophic lateral sclerosis (ALS) (38), Parkinson's disease (PD), Alzheimer's disease (AD) (39, 40), and so on. More interestingly, NF-H phosphoforms can be detected in the cerebrospinal fluid (CSF) and blood following experimental spinal cord and brain injury in rats (41). Consistent with the study of neurodegenerative diseases, our data from the AOH model showed early pNF-H accumulation in the soma of RGCs and ONH and the corresponding elevation of pNF-H in the aqueous humor. Unlike these data, the data from the COH model demonstrated that NF-H and its phosphoforms manifested general reduction and local disconnection in damaged axons. Hence, through a thorough study of the neurofilament reaction to acute and chronic damage, we enrich the pathology of glaucoma and creatively suggest both pNF-H accumulation and disconnection as biomarkers of RGC damage. However, limitations do exist in our study. First, although we observed a pathological change in NF-H same like tau in AD, we do not relate them together in current glaucoma models; since tau accumulation has been proven to promote neuronal degeneration in glaucoma (42), it is strongly believed that current neurofilament changes associated with glaucoma may share the same mechanism in AD. Second, the changes in pNF-H in the aqueous humor of a patient with glaucoma have been not further studied, so the role of pNF-H as a marker for disease progression remains unknown.

The histological study of the neurofilament in experimental glaucoma models truly helps us to learn more about the underlying damage of RGCs, but it poses a very practical question: can these be visualized *in vivo* by current techniques? Indeed, our clinical OCT imaging from patients with glaucoma indirectly demonstrates the different characteristics of the neurofilament between AOH and COH, but fails to provide many more detailed cellular changes. Benefit from the advance of ophthalmic imaging technology and labeling techniques, recently cellular-level RGC imaging, such as adaptive optics–optical coherence tomography (AO-OCT), has enabled the resolution of many cellular and subcellular structures (43, 44). Recent studies from AO-OCT in a patient with glaucoma have demonstrated subcellular changes, including enlarged RGC somas and nuclear hyporeflectivity, which is quite similar to the images of somatic NF-H or pNF-H accumulation in our study. Additionally, cytoskeleton alteration, including the neurofilament is found to be the major element contributing to the change in retinal nerve fiber layer birefringence, which serves as an early biomarker of glaucomatous damage prior to losses in thickness (45, 46). So, our experimental data have advanced our understanding of the latest findings observed in new techniques.

Combined with experimental and clinical data, our study revealed two distinct types of axonal degeneration. Insulted by acute damage, both proximal RGC cell bodies and the distal axon in the OHN are affected, which is characterized as intra-somatic and intra-axonal neurofilament compaction followed by fragmentation or degradation. While in COH, the neurofilament disconnection first begins distally and spreads toward the retina. Axonal degeneration is a common pathological event that occurs in various neurological diseases, but the cellular and molecular processes underlying different types of damage are highly variable. There are at least four forms of axonal degeneration in neurological diseases, the most common and classical two examples are Wallerian degeneration and dying back degeneration (retrograde degeneration) (47, 48). Wallerian degeneration is classically defined as the distal axons and cell body rapidly undergoing breaking down insult by an acute injury; dying back disorder is a neurodegenerative disorder in which axons die before cell bodies and/or in a retrograde pattern that begins with their distal ends. The damaged axons in our acute glaucoma seem like Wallerian degeneration; however, studies from some neurodegenerative diseases suggest that axonal degeneration associated with the accumulation of neurofilaments is distinct from a typical Wallerian degeneration (14). Importantly, studies of an RGC axotomy model showed that Wallerian and retrograde degeneration occurred synchronously, in which the neurofilament accumulated as well (49). Therefore, we believed that, in our AOH study, a combination of Wallerian and retrograde degeneration also presented. Conversely, the axons in COH degenerated like dying back degeneration. For the detailed reasons contributing to these phenomena, several proposed mechanisms including axonal transport failure, neurotrophic factors' deprivation, and ischemic and reperfusion damage would be used for explaining (50). Considering the degree and duration of elevated IOP in APACG, the reasons for acute damage would be a mixture of the aforementioned mechanisms, and neurotrophic factors' deprivation in the distal optic nerve may play the major role in chronic glaucoma.

The overall results of this study revealed early glaucomatous damage beyond the ONH, including peripheral RGC somatic NF-H, pNF-H aggregation in AOH, and distal optic nerve neurofilament disconnection in COH. These results also indicated the role of NF-H and pNF-H as biomarkers of glaucomatous damage. Furthermore, the two distinct neural degenerations existing between AOH and COH provide a concept that different neuroprotection strategies should be adopted in an angle-closed glaucoma treatment.

## Data availability statement

The original contributions presented in the study are included in the article/Supplementary material, further inquiries can be directed to the corresponding author.

## Ethics statement

The animal study was reviewed and approved by the Institutional Animal Care and Use Committee of Guang Dong Medical University and Zhongshan Ophthalmic Center. The studies involving human participants were reviewed and approved by the Ethics Committee of Huizhou Central People's Hospital. The patients/participants provided their written informed consent to participate in this study.

## Author contributions

Study design and drafting of the manuscript: LZ and HF. Acquisition of photographs: LZ and DL. Analysis and interpretation of data: GX and LZ. Critical revision of the manuscript: DW and LZ. Statistical analysis: LZ, ZC, and HF. All authors contributed to the article and approved the submitted version.
