# Supplementary material for: Alteration of neurofilament heavy chain and its phosphoforms reveals early subcellular damage beyond the optic nerve head in glaucoma
Source: Front Neurol. 2023 Mar 22;14:1091697. doi: 10.3389/fneur.2023.1091697 (PMC10073422; doi:10.3389/fneur.2023.1091697)
Supplement: Supplementary file 1 [file Data_Sheet_1.docx]

| Name | Company | Cat. No. |
| --- | --- | --- |
| COMPEL™ Magnetic, COOH Modified | Bangs Laboratories,Inc | UMC3F 10767 |
| Anti-neurofilament,Heavy | Millipore | AB5539 |
| Anti-neurofilament,Medium | Cell signaling technology | #2838 |
| Anti-neurofilament,Light | Millipore | MAB1615 |
| phosphrylated neuroﬁlament-H | Millipore | NE1022 |
| Anti-Brn3a | Millipore | MAB1585 |
| Anti-NeuN | Millipore | MABN140 |
| Phosphorylated Neurofilament (pNF-H) Sandwich ELISA Kit | Millipore | NS170 |

Supplement Figure 1. Table list of antibodies and kits
